# Enhanced electrical power generation using flame-oxidized stainless steel anode in microbial fuel cells and the anodic community structure

**DOI:** 10.1186/s13068-016-0480-7

**Published:** 2016-03-12

**Authors:** Takahiro Yamashita, Mitsuyoshi Ishida, Shiho Asakawa, Hiroyuki Kanamori, Harumi Sasaki, Akifumi Ogino, Yuichi Katayose, Tamao Hatta, Hiroshi Yokoyama

**Affiliations:** Animal Waste Management and Environment Division, NARO Institute of Livestock and Grassland Science2, Ikenodai, Tsukuba, 305-0901 Japan; Faculty of Agriculture, Utsunomiya University, 350 Minemachi, Utsunomiya, 321-8505 Japan; Agrogenomics Research Center, National Institute of Agrobiological Sciences (NIAS), 1-2 Owashi, Tsukuba, 305-8634 Japan; Biological Resources and Post-harvest Division, Japan International Research Center for Agricultural Sciences, 1-1 Owashi, Tsukuba, 305-8686 Japan

**Keywords:** Community structure, Energy recovery, Flame oxidation, *Geobacter*, Microbial fuel cell, Stainless steel, Wastewater treatment

## Abstract

**Background:**

Carbon-based materials are commonly used as anodes in microbial fuel cells (MFCs), whereas metal and metal-oxide-based materials are not used frequently because of low electrical output. Stainless steel is a low-cost material with high conductivity and physical strength. In this study, we investigated the power generation using flame-oxidized (FO) stainless steel anodes (SSAs) in single-chambered air-cathode MFCs. The FO-SSA performance was compared to the performance of untreated SSA and carbon cloth anode (CCA), a common carbonaceous electrode. The difference in the anodic community structures was analyzed using high-throughput sequencing of the V4 region in 16S rRNA gene.

**Results:**

Flame oxidation of SSA produced raised node-like sites, predominantly consisting of hematite (Fe_2_O_3_), on the surface, as determined by X-ray diffraction spectroscopy. The flame oxidation enhanced the maximum power density (1063 mW/m^2^) in MFCs, which was 184 and 24 % higher than those for untreated SSA and CCA, respectively. The FO-SSA exhibited 8.75 and 2.71 times higher current production than SSA and CCA, respectively, under potentiostatic testing conditions. Bacteria from the genus *Geobacter* were detected at a remarkably higher frequency in the biofilm formed on the FO-SSA (8.8–9.2 %) than in the biofilms formed on the SSA and CCA (0.7–1.4 %). Bacterial species closely related to *Geobacter metallireducens* (>99 % identity in the gene sequence) were predominant (93–96 %) among the genus *Geobacter* in the FO-SSA biofilm, whereas bacteria with a 100 % identity to *G. anodireducens* were abundant (>55 %) in the SSA and CCA biofilms.

**Conclusions:**

This is the first demonstration of power generation using an FO-SSA in MFCs. Flame oxidation of the SSA enhances electricity production in MFCs, which is higher than that with the common carbonaceous electrode, CCA. The FO-SSA is not only inexpensive but also can be prepared using a simple method. To our knowledge, this study reveals, for the first time, that the predominant *Geobacter* species in the biofilm depends on the anode material. The high performance of the FO-SSA could result from the particularly high population of bacteria closely related to *G. metallireducens* in the biofilm.

**Electronic supplementary material:**

The online version of this article (doi:10.1186/s13068-016-0480-7) contains supplementary material, which is available to authorized users.

## Background

Microbial fuel cells (MFCs) are prospective bioreactors that generate electricity as well as purify wastewater [1]. Under anaerobic conditions, bacteria decompose the organic matter in wastewater to CO_2_ by redox reactions in the MFCs. The electrons generated in these redox reactions are transferred to the anode by the bacteria, and flow to the cathode via an external circuit. In air-cathode single-chambered MFCs [2], the electrons react with oxygen in the air on the cathode. As electron transfer to the anode is the key, the development of anode materials to facilitate the reaction is imperative for improving the power output of MFCs. Carbon-based anodes such as carbon cloth, carbon brush, and carbon felt have been employed in most studies on MFCs [3]. In addition, sophisticated carbonaceous anodes have been reported including carbon nanotubes [4], modified graphene [5], and carbon felt with a catalyst [6]. Carbonaceous materials are conductive and chemically stable, in addition to exhibiting large effective surface areas and high biocompatibility to microbes. Metal and metal-oxide-based anodes are not typically used because of low power generation and biocompatibility in the MFCs [3]. Recently, an anode made of stainless steel (SS) foam has been reported to produce high current density in bioelectrochemical systems (BESs) [7]. The coating of the SS anode (SSA) with graphene has been demonstrated to increase the current density in MFCs [8]. Moreover, the flame oxidation of SSA has been reported to enhance current output in BESs [9]. Flame oxidation leads to the formation of iron oxide nanoparticles on the SS surface, and is likely to increase the biocompatibility.

*Geobacter* species are representative electricity-generating bacteria [10] and are called exoelectrogens [11]. This species reduces insoluble iron oxide coupled with acetate oxidation under anoxic environments [12]. c-type cytochromes in the extracellular matrix of *Geobacter* species are involved in electron transfer to Fe(III) oxide [13]. *Geobacter sulfurreducens* forms electrically conductive pili, through which electron transfer to Fe(III) oxide is suggested to take place [14]. Currently, 21 species including two subspecies have been identified in the genus *Geobacter*. However, thus far, it is unclear as to what types of electrode materials (as extracellular electron acceptors) are preferred by each of the species, and whether the preference to electrode materials differs among the species. Next-generation sequencing technology is a powerful tool for analyzing the bacterial community structure at extremely fine resolution [15]. High-throughput sequencing analyzes multimillion reads for the 16S rRNA gene, by which slight differences among bacterial community structures can be detected.

SS is a low-cost material that is highly conductive compared to standard carbonaceous electrodes. SS has sufficient chemical and mechanical strength, and is easy to form into a desired shape. These properties of SS render it suitable for the construction of large electrodes at low cost. Current production using flame-oxidized (FO) SSAs in BESs has been reported [9]. However, power generation using FO-SSA in MFCs has not been examined. In contrast to that in the BESs, the electrode potential of anodes in the MFCs is not controlled to be constant and fluctuates during cultivation, exhibiting dependence on environmental factors such as substrate concentration, pH, and oxygen intrusion. Hence, the usefulness of FO-SSA for power generation in MFCs is unclear. Furthermore, information regarding the types of bacteria that preferentially form biofilms on the FO-SSA surface is unknown. In this study, the power density and current production by MFCs containing FO-SSA are evaluated and compared with those containing a carbon cloth anode (CCA), which is one of the common carbonaceous anodes. Furthermore, bacterial community structures developed on both the anodes are characterized in detail by next-generation sequencing.

## Results and discussion

### Surface characteristics of FO-SSA

**Fig. 1:**
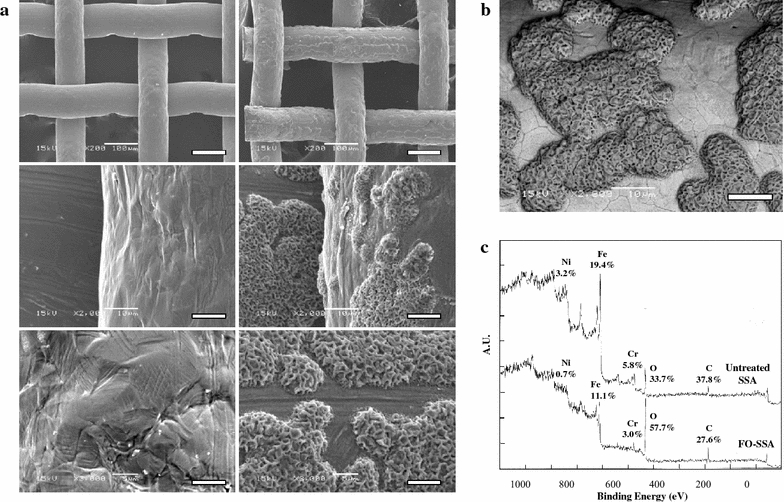
Change in the SSA surface caused by flame oxidation. **a** SEM images of untreated SSA (*left*) and FO-SSA (*right*). The *scale bars* represent 100 μm (*top*), 10 μm (*middle*), and 5 μm (*bottom*). **b** BEC image of FO-SSA. The *bar* represents 10 μm. **c** XPS profiles of untreated SSA and FO-SSA

**Fig. 2:**
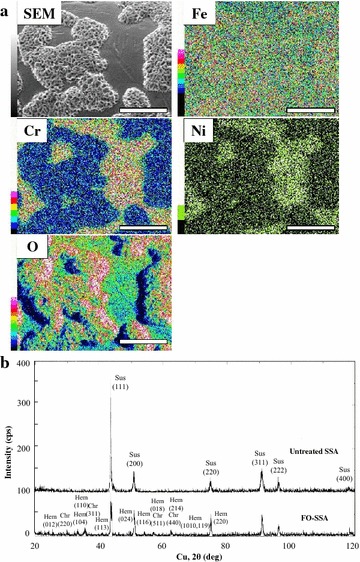
Characterization of the FO-SSA surface. **a** SEM–EDS images of FO-SSA for each atom. *Bars* represent 20 μm. **b** XRD profiles of untreated SSA and FO-SSA. *Sus* SUS304 (Cr, 0.19; Fe, 0.7; Ni, 0.11), *Hem* hematite, *Chr* chromite

Flame oxidation led to increase in the weight of SSA by 0.19 mg/cm^2^ and the color of SSA changed to black. The conductivity of FO-SSA was retained after flame oxidation, whereas the resistance value of FO-SSA ranged widely from <1 to 100 Ω/cm, depending on the analysis sites. Scanning electron microscopy (SEM) analysis revealed the formation of many raised node-like sites (5–200 μm in length, 10–50 μm in width, and 0.5–3 μm in height) on the FO-SSA surface (Fig. 1a). In the backscattered electron composition (BEC) analysis, the raised sites exhibited a darker contrast compared to the flat region (Fig. 1b). Typically, in BEC analyses, heavy atoms such as metal atoms exhibit a lighter contrast, whereas light atoms such as carbon and oxygen appear darker. Thus, it is suggested that the percentage of light atom(s) was increased in the raised sites present on the FO-SSA surface. X-ray photoelectron spectroscopy (XPS) analysis was conducted for measuring the atomic composition of the FO-SSA surface. By flame oxidation, the percentage of oxygen increased from 33.7 to 57.7 % (Fig. 1c). Owing to this increase, the percentages of the other atoms relatively decreased. The Fe/Cr/Ni ratio changed from 1.00:0.30:0.16 to 1.00:0.27:0.06. In other words, the Fe and Cr ratios did not significantly change, whereas the Ni ratio decreased. The presence of carbon was detected, which was probably caused by the adsorption of adventitious materials. In the narrow-range XPS spectra (Additional file 1: Figure S1 A, B), the Fe and Cr peaks were shifted to the high-binding energy sides as a result of flame oxidation, suggesting the oxidation of Fe and Cr. After flame oxidation, the intensity of the Ni photopeak was below the detection level in the XPS analysis (Additional file 1: Figure S1C). SEM energy-dispersive spectroscopy (EDS) was employed to investigate the difference in the atomic composition between the raised sites and flat areas on the FO-SSA surface in more detail (Fig. 2a). Uniform distribution of Fe was observed in both the regions. Cr was more abundantly present in the flat areas compared to the raised sites. Ni was weakly detected and not detected in the flat areas and raised sites, respectively. Oxygen was concentrated in the raised sites, particularly at the apex of the raised sites, and was also present in the flat areas at reduced levels. Thus, these images indicate that the raised sites on the FO-SSA surface are mainly composed of iron oxide, whereas the flat areas contain iron and chromium oxides. The FO-SSA surface was further analyzed by X-ray diffraction (XRD) for determining the molecular composition (Fig. 2b). In the XRD profile of untreated SSA, clear peaks were detected for SUS304 (Cr, 0.19; Fe, 0.7; Ni, 0.11). In the case of FO-SSA, signals for hematite (Fe_2_O_3_) and chromite (FeCr_2_O_4_) were observed, while no peak was observed for Fe_3_O_4_ or Ni. In summary, these results suggest that the raised sites on the FO-SSA surface are mainly composed of hematite, whereas the flat areas contain both hematite and chromite.

### Power density and current production of FO-SSA

**Fig. 3:**
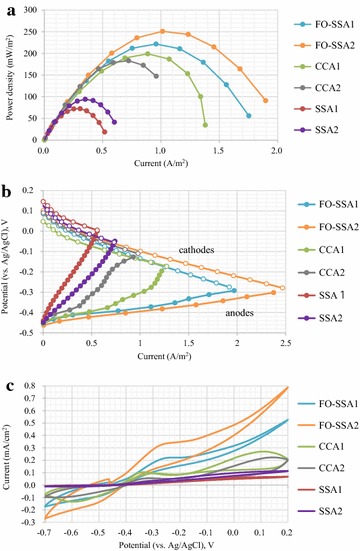
Comparison of FO-SSA with CCA and untreated SSA in MFCs equipped with membranes. The peptone medium was used as the feedstock for the MFCs. Power density (**a**), polarization curves for the anodes and cathodes (**b**), and CV profiles (**c**) are shown. In **b**, data for the anodes and corresponding cathodes are represented as *solid* and *clear circles*, respectively

FO-SSAs (FO-SSA1 and FO-SSA2) were installed in the air-cathode single-chambered MFCs equipped with the membrane. To precisely compare the anode performance, CCAs (CCA1 and CCA2) with identical thickness and untreated SSA (SSA1 and SSA2) were utilized. A total of six MFCs were operated using a peptone medium. After culture for approximately 8 weeks, the MFCs with FO-SSA and SSA reached a plateau in terms of electricity generation, as judged by manual periodical measurement of polarization curves. The MFCs with CCAs took a shorter period (4 weeks) to reach stable performance. A thick biofilm was formed on the surfaces of all the anodes, as observed by SEM (Additional file 2: Figure S2). The MFCs were operated for 150 days during which no decrease in electricity generation was observed (Additional file 3: Figure S3). This result suggests the long-term stability of FO-SSA in the MFCs. The flame oxidation of SSA enhanced the maximum power density in the MFCs. MFCs containing FO-SSA generated 221–251 mW/m^2^ of maximum power density on average, which was 184 and 24 % higher than untreated SSA and CCA, respectively (Fig. 3a). The internal resistance of the MFCs with FO-SSA was the lowest (92–101 Ω), followed by those of MFCs with CCA (111–151 Ω) and untreated SSA (307–381 Ω). Coulombic efficiency [16] of the MFCs with FO-SSA (15 %–21 %) and CCA (15 %–17 %) was at similar values. FO-SSA exhibited the highest current response in the potentiostatic tests (Fig. 3b). At a potential of −0.3 V (vs. Ag/AgCl), the current generated by FO-SSA was 1.8–2.4 A/m^2^, which was 8.75 and 2.71 times higher than those of untreated SSA and CCA, respectively. In cyclic voltammetry (CV) analysis (Fig. 3c), the highest current response was also observed in FO-SSA at all potentials from −0.7 V to 0.2 V (vs. Ag/AgCl). The overall CV curves for all the anodes were similar in shape. An abiotic control experiment was set up for all anodes, in which CV analysis was performed without the inoculation of bacteria. The abiotic controls produced negligible response currents (Additional file 4: Figure S4).

**Fig. 4:**
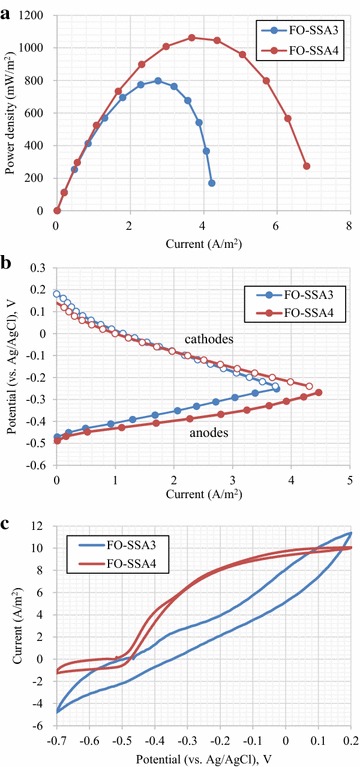
Electricity generation by FO-SSA in membrane-less MFCs. MFCs with FO-SSA3 and FO-SSA4 were fed with the peptone and acetate media, respectively. Power density (**a**), polarization curves for the anodes and cathodes (**b**), and CV profiles (**c**) are shown. In **b**, data for the anodes and corresponding cathodes are represented as *solid* and *clear circles*, respectively

Usually, the power output by membrane-less MFCs is higher than that of MFCs equipped with a membrane, since the resistance of the membrane is absent in the former case [17]. To further evaluate the performance of FO-SSA, testing was conducted using membrane-less air-cathode MFCs. The peptone and acetate medium were fed to the MFCs equipped with FO-SSA3 and FO-SSA4, respectively. After culture for 5 weeks, the MFCs showed stable electricity generation. The maximum power density with the acetate medium (1063 mW/m^2^) was 33 % higher than that with the peptone medium (Fig. 4a). In the tests conducted under potentiostatic conditions, a current density of 3.92 A/m^2^ was generated in the acetate medium at a potential of −0.3 V (vs. Ag/AgCl), which was 36 % higher than the current density generated in the peptone medium (Fig. 4b). In the CV analysis (Fig. 4c), FO-SSA4 showed higher current response than FO-SSA3 in the potential range from −0.5 to 0 V (vs. Ag/AgCl), which encompassed the typical working potential for the anode in MFCs. These results show that acetate is superior to peptone as the feedstock for MFCs with FO-SSA. In the case of MFCs with carbonaceous anodes, acetate media have been reported to give rise to relatively high maximum power densities [18].

This is the first report demonstrating that the flame oxidation of SSA remarkably enhances power and current production in MFCs. In the present study, identical thickness and projected surface area of the anodes were used for comparative purposes, and the MFCs were operated under identical culturing conditions. This study clearly demonstrates that the FO-SSA is relatively superior to the common carbonaceous electrode (CCA) in terms of power and current generation; however, bacteria require additional time for acclimation in order to stably produce electricity in the former. MFC studies have been conducted under various conditions, including various reactor configurations, medium compositions, and cathode types, all of which affect the power densities in MFCs. More importantly, MFC outputs are usually not scalable [19]. In other words, small-sized MFC reactors tend to produce higher power densities than large-sized reactors. These factors should be considered when comparing the data in this study with values reported in the literature. The reported maximum power density values using single-chambered air-cathode MFCs with a reactor volume more than 100 cm^3^, fed with defined substrates, are predominantly in the range from 400 to 1200 mW/m^2^ [1, 20]. The maximum power density of 1063 mW/m^2^ obtained in the membrane-less MFC with the FO-SSA (volume: 125 mL) is at the higher end of the range, showing the usefulness of the FO-SSA. While sophisticated carbonaceous anodes such as carbon nanotubes produce high power densities in small-sized MFC reactors, these anodes are expensive, and the preparation methods are complicated, making them unsuitable for the construction of large-scale electrodes. In contrast, the FO-SSA is inexpensive and physically strong, and the preparation procedure is rapid and simple, involving simply toasting with a flame, allowing it to be used for large-scale construction. Based on these characteristics, the FO-SSA is useful for practical applications, particularly for scaled-up MFC reactors.

### Community structure of the anode biofilms

Acetate is the preferred substrate for the *Geobacter* species, and thus, *Geobacter*-dominant (>50 %) biofilms can be readily developed on anodes by using the acetate medium. However, actual wastewater such as sewage consists of complex substrates, and contains large amounts of non-exoelectrogenic bacteria. The formation of exoelectrogen-dominant biofilms on anodes is challenging in practice, when a medium other than acetate is fed to the MFCs. Therefore, the effect of flame oxidation on the bacterial community structure was examined using MFCs fed with the peptone medium. FO-SSAs (FO-SSA5 and FO-SSA6), CCA3, and untreated SSA3 were incorporated into MFCs equipped with membranes. For comparison, an FO-SSA with an open-circuit operation (FO-SSA7-o.c) and activated sludge (AS) inoculated into the MFC reactors were also analyzed. Additional file 5: Table S1 summarizes the operational taxonomic unit (OTU) distribution and alpha diversity of the communities. The alpha diversity indices suggest that the diversities of all the anodic communities are at comparable levels, and they are lower than those of AS. The slopes of the rarefaction curves for the anodic communities were also similar (Additional file 6: Figure S5A). In the beta diversity analysis using the UniFrac method [21], the anodic communities under closed-circuit operation (FO-SSA5, FO-SSA6, SSA3, and CCA3) were clustered together in the plot (Additional file 6: Figure S5B), and were located at a distance from AS and FO-SSA7-o.c. These results indicate that the overall community structures of the anodic biofilms under closed-circuit operation are similar to each other, despite the difference in the anode materials, and are distinct from the community operated under open-circuit conditions.

**Fig. 5:**
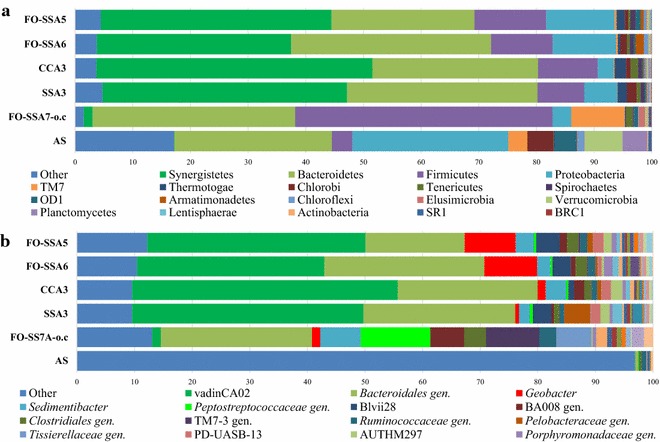
Bacterial community structures in the anodic biofilms and AS (inoculum) at the phylum (**a**) and genus (**b**) levels, as analyzed using the 16S rRNA gene

Synergistetes, bacteroidetes, firmicutes, and proteobacteria were the predominant phyla in the anodic biofilms under closed-circuit operation (Fig. 5a). The three phyla proteobacteria, firmicutes, and bacteroidetes are frequently observed in MFCs [22]. In this study, synergistetes was significantly more abundant in the communities under closed-circuit operation (33.7–47.9 %) compared to the FO-SSA7-o.c community (1.5 %). Additional file 7: Table S2 lists the genera detected in FO-SSA5 and FO-SSA6 with higher abundance than in FO-SSA7-o.c. Among these genera, the candidate genus vadinCA02 in the phylum synergistetes exhibited the highest changes in frequency (Fig. 5b). Bacteria affiliated to vadinCA02 showed a 83–90 % identity to *Aminomonas paucivorans*, *Synergistes jonesii,* and *Cloacibacillus porcorum* in the phylum synergistetes in terms of the V4 region of the 16S rRNA gene sequence. The members of the phylum synergistetes include non-saccharolytic anaerobes, which degrade amino acids to acetate [23]. The population of Blvii28 was higher in FO-SSA5, FO-SSA6, and SSA3 (3.2–4.1 %) than in CCA3 (1.0 %) and FO-SSA7-o.c (<0.1 %), suggesting that the bacteria assigned to Blvii28 have a preference for both flamed and untreated SSAs rather than CCA. The bacteria assigned to Blvii28 showed a 86–87 % identity to *Parabacteroides distasonis* of the phylum Bacteroidetes. *Parabacteroides* species are obligately anaerobic bacteria that degrade saccharides, forming acetate and succinate as major end products [24]. These observations imply that bacteria assigned to vadinCA02 and Blvii28 decompose peptone and saccharides into acetate, respectively, and the acetate thus produced may serve as a fuel for the generation of current for exoelectrogens.

### Preference of *Geobacter* species to FO-SSA

A putative electrogenic genus that prefers FO-SSA to CCA and untreated SSA was searched with the following criteria: (1) detection frequency is greater than 0.1 % in FO-SSA5 and FO-SSA6 and (2) the frequencies in FO-SSA5 and FO-SSA6 are over three times higher compared to CCA, untreated SSA, and FO-SSA7-o.c. Surprisingly, only one genus *Geobacter* was found to satisfy these criteria. The detection frequency of the *Geobacter* species in FO-SSAs (8.8–9.2 %) was greater by over six times compared to those of CCA, untreated SSA, and FO-SSA7-o.c (0.7–1.4 %). Interestingly, the species distribution in the genus *Geobacter* was considerably different among the anodic communities (Table 1). Bacteria closely related to *G. metallireducens* (>99 % identity) were predominant in FO-SSA5 and FO-SSA6 with a frequency of 93.6–96.5 % (number of reads assigned to the species per number of reads assigned to the genus *Geobacter*). Bacteria with a 100 % identity to *G. anodireducens* were abundant in both CCA3 and SSA3 (54.7–66.9 %). Bacteria with >99 % identity to *G. chapelleii* were exclusively detected (99.7 %) in FO-SSA7-o.c. These observations show that bacteria found to be closely related to *G. metallireducens* prefer FO-SSA over CCA and untreated SSA. The very low frequency of these bacteria in FO-SSA7-o.c (0.1 %) indicates that the flow of electrons in FO-SSA is needed for growth. This implies that these bacteria might transfer electrons to FO-SSA via the raised iron oxide sites, since the raised sites are present only on the FO-SSA surface.

**Table 1:** Distribution of the *Geobacter* species in anodic biofilms

Most closely related species	FO-SSA5	FO-SSA6	CCA3	SSA3	FO-SSA7-o.c
*Geobacter metallireducens*	96.5	93.6	1.4	6.4	0.1
*G. pickeringii*	2.0	1.0	8.9	17.7	0.0
*G. anodireducens*	0.8	0.2	54.7	66.9	0.0
*G. thiogenes*	0.4	1.2	1.1	0.4	0.1
*G. daltonii*	0.2	0.8	8.1	7.9	0.0
*G. chapelleii*	0.1	1.6	20.4	0.6	99.7
*G. pelophilus*	0.0	1.6	5.4	0.2	0.0

Values indicate the percentage of the number of reads assigned to the species per number of reads assigned to the genus *Geobacter*

*Geobacter anodireducens* [25] is a close relative of *G. sulfurreducens* [26] with their 16S rRNA gene sharing 98 % identity. *G. sulfurreducens* is commonly enriched from environmental samples in MFC cultures when carbon-based anodes are used. While *G. sulfurreducens* and *G. anodireducens* reduce Fe(III) with H_2_, *G. metallireducens* [12] and *G. chapelleii* [27] do not. Compared to *G. metallireducens*, *G. sulfurreducens* has been reported to produce higher current in a microbial electrolysis cell equipped with a carbonaceous anode [28]. Although individual signatures of *Geobacter* species have been well examined with respect to substrate utility and metal-oxide reduction, the preference of *Geobacter* species to electrode materials has not been investigated. This study is the first report revealing the dependence of the predominant *Geobacter* species in the biofilm on the anode material. Bacteria closely related to *G. metallireducens* prefer FO-SSA over CCA and untreated SSA as the terminal electron acceptor, whereas bacteria with high similarity to *G. anodireducens* prefer both CCA and untreated SSA. The formation of exoelectrogen-dominant biofilms on the anode surface is crucial for attaining high electrical output from actual wastewater. The population of *Geobacter* species was much lower in the biofilm developed on the carbon-based electrode CCA when the peptone medium was used as the feedstock. This study has shown that the flame oxidation of SSA can increase the population of bacterial species closely related to *G. metallireducens* in the anode biofilm under non-acetate feeding conditions. The results of the current study suggest that the high performance of FO-SSA in MFCs is achieved by the increased population of bacteria closely related to *G. metallireducens*.

## Conclusions

Flame oxidation of SSA increases the power and current production in MFCs. The maximum power density of MFCs with FO-SSA is higher than that of MFCs with conventionally used carbonaceous electrode, CCA. The population of the *Geobacter* species is more abundant in the FO-SSA biofilm compared to untreated SSA and CCA biofilms. The bacterial species closely related to *G. metallireducens* prefer FO-SSA over SSA and CCA. The increased population of these bacteria is suggested to lead to the high performance of FO-SSA. FO-SSA is inexpensive and is easily prepared, rendering it suitable for large-scale applications in MFCs.

## Methods

### Flame oxidation of SS and MFC operation

The SS used (Nilaco, Tokyo, Japan) was a 0.2-mm-thick mesh (100 mesh, SUS304, wire diameter of 100 μm). The mesh was anchored with tweezers, and was flamed (>1200 °C) for 10 min with a kitchen stove-top burner using natural gas as the fuel, with the flame strength set to the maximum level. The mesh was placed in the blue outer zone (hottest part) of the flame, and was turned inside out every 2 min. In a comparison experiment, the FO-SS mesh (5 cm × 5 cm) was placed on one side of a cubic MFC reactor with an inner volume of 125 mL (5 cm × 5 cm × 5 cm). The reactor had an air-cathode single-chambered configuration, fabricated with 0.8-cm thick polycarbonate resin. The air-cathode, which was placed opposite to the anode, was composed of a carbon cloth with 0.5 mg/cm^2^ of Pt catalyst. The air-cathode was fused with a cation-exchange membrane Selemion HSF (AGC Engineering, Chiba, Japan). The reactor was filled with the peptone medium (pH 7.0–7.2) containing (per liter of distilled water) 2 g peptone, 1 g meat extract, 0.3 g urea, 0.6 g NaH_2_PO_4_·2H_2_O, 2 g NaHCO_3_, 0.12 g NaCl, 0.05 g KCl, 0.03 g CaCl_2_·2H_2_O, and 0.05 g MgSO_4_·7H_2_O. The MFC was operated at 25 °C in a fed-batch mode. AS, collected at a livestock-wastewater treatment plant of the NARO Institute of Livestock and Grassland Science, Tsukuba, Japan, was inoculated into the MFC as seed sludge. For comparison, 0.2-mm-thick CCA and non-flamed SSA were used in the cubic reactors. The MFCs were connected to a 4.3 kΩ external resistor, and the resistance value was decreased stepwise during operation.

In the experiment using the membrane-less air-cathode MFCs, the FO-SS mesh (4 × 80 cm) was folded and placed in the cubic reactors, which were equipped with a carbon paper cathode without a membrane. The MFCs were fed with the peptone medium or the acetate medium, and were operated at 30 °C. The acetate medium had the same composition as the peptone medium, except that peptone was replaced with sodium acetate.

### Electrode surface characterization

Additional file 8: Table S3 lists the atomic composition of the SS used, as measured by X-ray fluorescence spectrometry (XRF). The surface topography of FO-SSA was characterized by SEM using a JSM-5600LV (JEOL, Tokyo, Japan) instrument operated at 15 kV, followed by EDS. BEC images were recorded using this instrument. XPS was employed for investigating the electronic state of FO-SSA. XPS spectra were recorded on a VG-Scienta ESCA-300 system (Uppsala, Sweden) with a monochromatic AlKα X-ray source (*hv* = 1486.6 eV) at a power of 1.0 kW and base pressure of 7.3 × 10^−8^ Pa in the analytical chamber. Electrostatic charging caused by the poor electrical conductivity of the samples was minimized using a flood gun. Using a takeoff angle of 90°, wide scans were performed for identifying C, O, Cr, Fe, and Ni. Molecules on the electrode surface were analyzed by XRD. FO-SSA directly placed on a glass holder was measured using RAD-X (Rigaku Co., Tokyo, Japan) under the following conditions: CuKα, 40 kV; 25 mA; divergence slit, 1°; anti-scatter slit, 1°; receiving slit, 0.3 mm; monochromator slit, 0.6 mm; scan rate, 2°/min; and scan step, 0.02°.

### Electrochemical analysis

The polarization curve for the MFCs was obtained by recording the current response to potential decrease in steps of 50 mV using a potentiostat/galvanostat (AutoLab PGSTAT12; Metrohm Autolab, Utrecht, Netherlands). Each potential value was set for 50 s, and the data at the last time points were collected at each potential in order to allow for current stabilization. Electrical power (*P* = IV) was calculated from the measured current (I) and set potential (V), and power density was normalized with respect to the projected-cathode area (m^2^). The internal resistance of the MFCs was calculated from the slope of the polarization curve for the MFC reactors [16]. The polarization curves for each electrode were recorded by changing the electrode potential in steps of 20 mV. A Pt-coated counter electrode and an Ag/AgCl reference electrode were used in this setup and each potential value was set for 20 s. CV of the anodes was conducted at a scan rate of 3 mV/s in a potential window from −0.7 to 0.2 V (vs. Ag/AgCl) using the potentiostat. Coulombic efficiency was estimated from the amount of electron flow and decrease in the chemical oxygen demand of the medium [16].

### Community structure analysis

Next-generation sequencing was performed by the MiSeq Illumina sequencing platform (Illumina Inc., CA, USA) using the V4 region of the 16S rRNA gene [15]. The cubic MFCs with the membrane, fed with the peptone medium, were operated at 25 °C. The MFCs reached a stable performance after 2 months, and were cultured for a further 2-month period. The biofilms formed on the anodes were extensively washed with distilled water, and the genomic DNA of the bacteria tightly adhering to the anodes was extracted using an UltraClean™ Soil DNA Isolation kit (Mo Bio Laboratories, Carlsbad, CA, USA). Libraries were constructed from the bacterial genomic DNA by polymerase chain reaction using 563F and 802R primers, which included the Illumina overhang adapter sequences, according to the manufacturer’s instructions. The libraries were sequenced on 300PE MiSeq run, and paired-end read data were processed with the QIIME software [29]. The read sequences were joined, quality-checked, and clustered into OTUs by the Uclust method [30]. After chimera check, taxonomic classification, rarefaction curves, and alpha diversity indices were computed with QIIME. Beta diversity analysis was calculated using a weighted UniFrac distance matrix [21], and the result was visualized with a principal coordinate (PCo) plot. Taxonomic assignment to the *Geobacter* species was conducted by a BLAST search at a similarity threshold of 97 %.

## Additional files

10.1186/s13068-016-0480-7 XPS narrow spectra of Fe*2p* (A), Cr*2p* (B), and Ni*2p* (C) for FO-SS and untreated SS.
10.1186/s13068-016-0480-7 SEM images of the biofilms binding to FO-SSA, untreated SSA, and CCA. The bars represent 0.1 mm.
10.1186/s13068-016-0480-7 Time courses of electricity generation in MFCs equipped with FO-SSA or CCA.
10.1186/s13068-016-0480-7 CV profiles of FO-SSA, CCA, and untreated SSA with or without inoculation of bacteria.
10.1186/s13068-016-0480-7 Number of reads after chimera check and alpha diversity analysis of anodic communities in MFCs.
10.1186/s13068-016-0480-7 Rarefaction curves (A) and PCo plot (B) showing the relationship among the bacterial communities of the anodic biofilms and AS inoculated into the MFCs.
10.1186/s13068-016-0480-7 Genera detected in the biofilms of FO-SSA5 and FO-SSA6 with higher abundance than those in FO-SS7-o.c.
10.1186/s13068-016-0480-7 Atomic composition of SS used in this study.

## Abbreviations

AS: activated sludge
BEC: backscattered electron composition
BES: bioelectrochemical system
CCA: carbon cloth anode
CV: cyclic voltammetry
EDS: energy-dispersive spectroscopy
FO: flame oxidized
MFC: microbial fuel cell
o. c: open circuit
OTU: operational taxonomic unit
PCo: principal coordinate
SEM: scanning electron microscopy
SS: stainless steel
SSA: stainless steel anode
XPS: x-ray photoelectron spectroscopy
XRD: x-ray diffraction
XRF: x-ray fluorescence spectrometry
