# Supplementary figures and images for: Enhanced electrical power generation using flame-oxidized stainless steel anode in microbial fuel cells and the anodic community structure
Source: Biotechnol Biofuels. 2016 Mar 12;9:62. doi: 10.1186/s13068-016-0480-7 (PMC4788886; doi:10.1186/s13068-016-0480-7)

**A**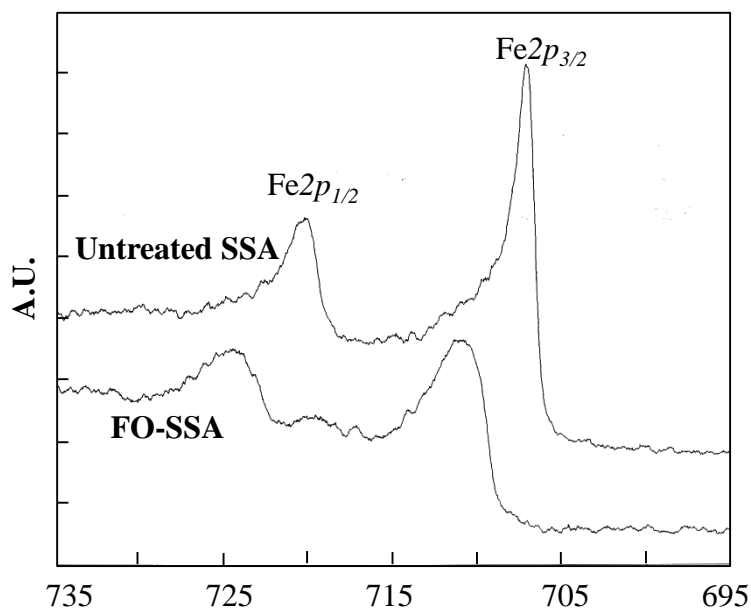**B**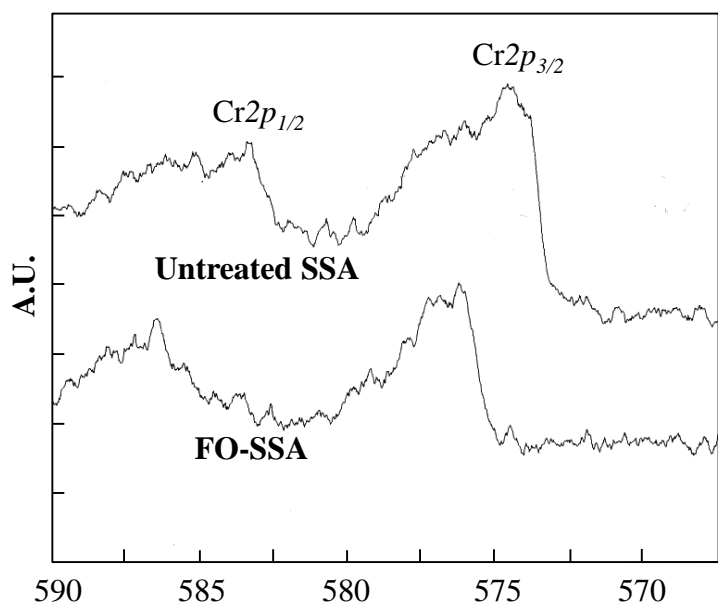**C**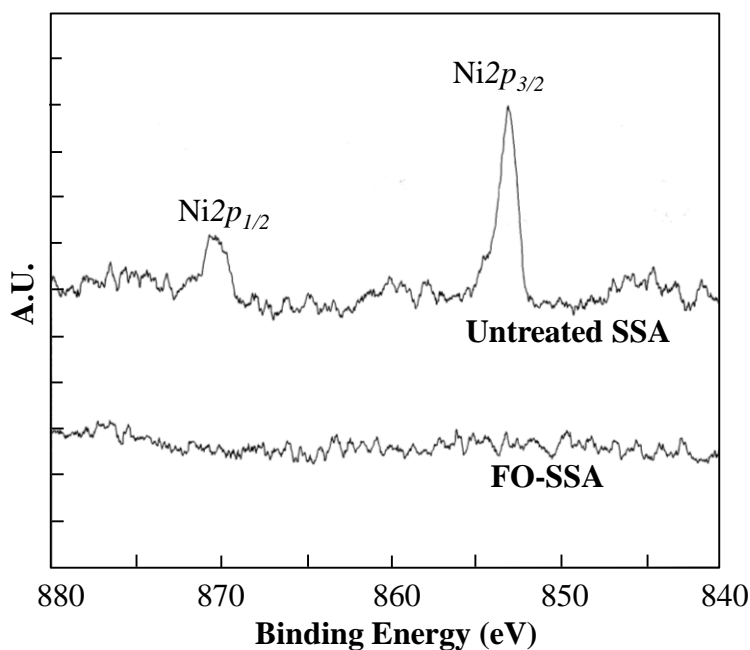

**Figure S1. XPS narrow spectra of Fe2p (A), Cr2p (B), and Ni2p (C) for FO-SSA and untreated SSA.**

Supplement: Supplementary file 1 — 10.1186/s13068-016-0480-7 XPS narrow spectra of Fe2p (A), Cr2p (B), and Ni2p (C) for FO-SS and untreated SS. [file 13068_2016_480_MOESM1_ESM.pdf]

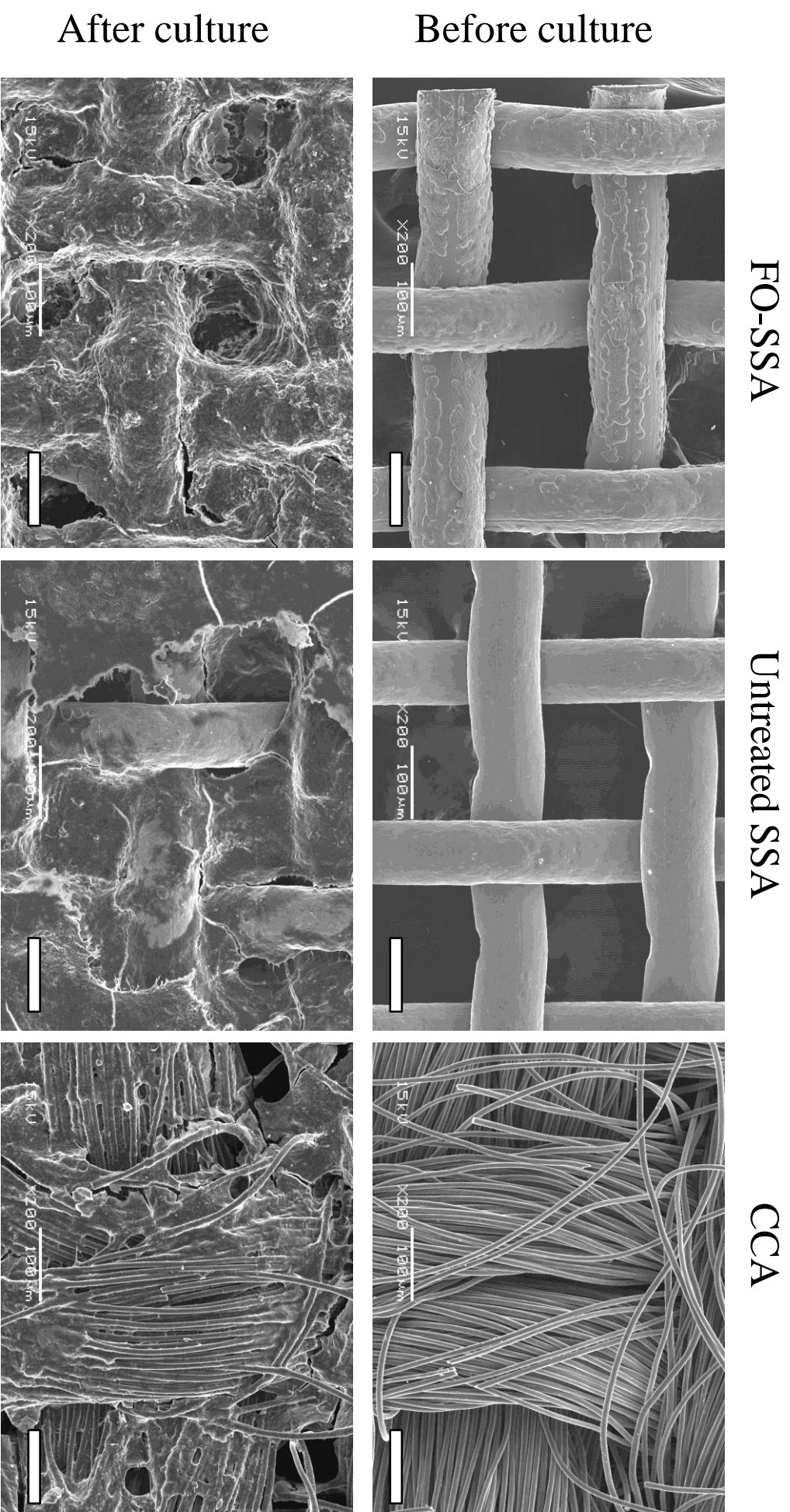

**Figure S2. SEM images of the biofilms binding to FO-SSA, untreated SSA, and CCA. The bars represent 0.1 mm.**

Supplement: Supplementary file 2 — 10.1186/s13068-016-0480-7 SEM images of the biofilms binding to FO-SSA, untreated SSA, and CCA. The bars represent 0.1 mm. [file 13068_2016_480_MOESM2_ESM.pdf]

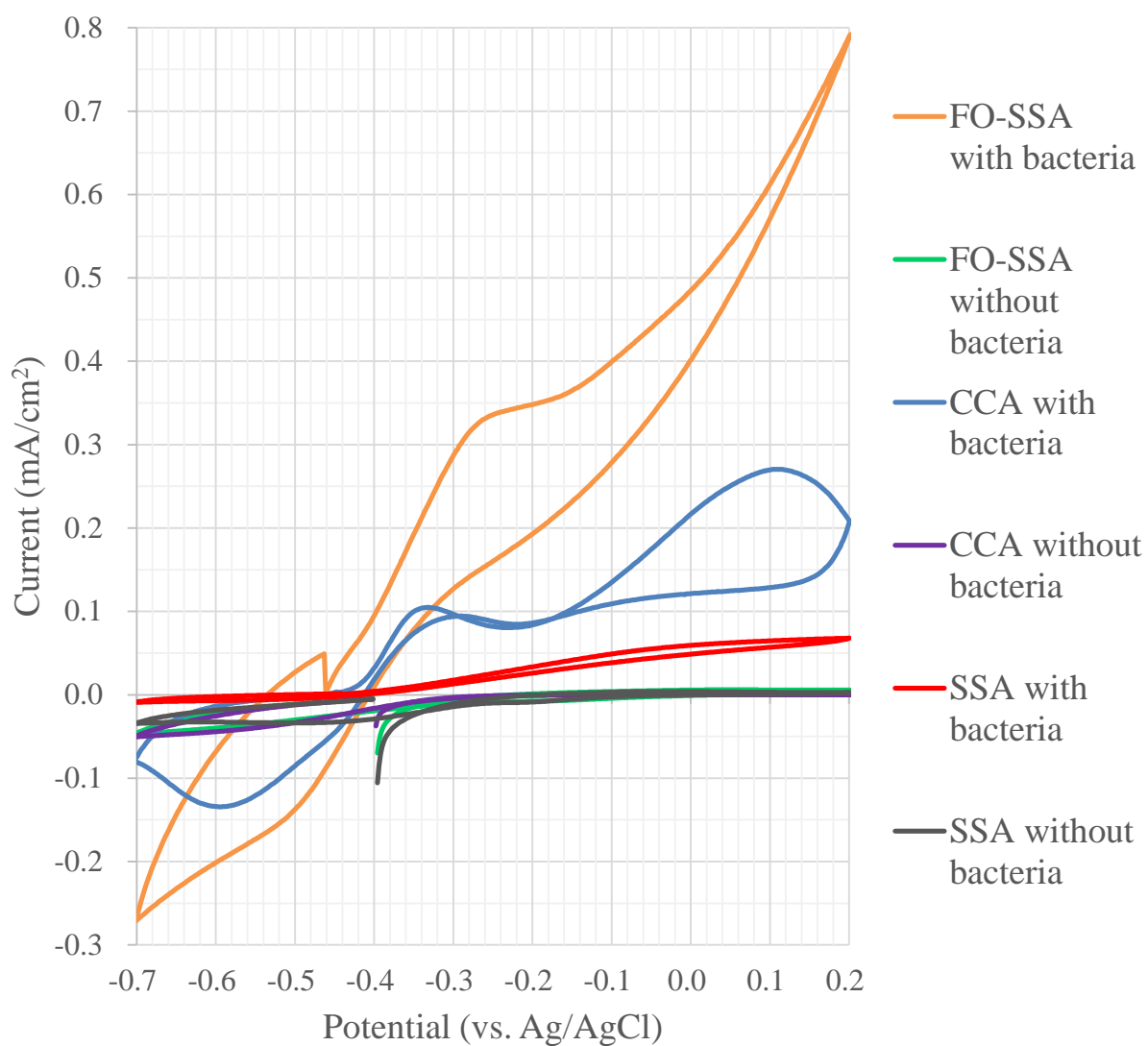

**Figure S4. CV profiles of FO-SSA, CCA, and untreated SSA with or without inoculation of bacteria.**

Supplement: Supplementary file 4 — 10.1186/s13068-016-0480-7 CV profiles of FO-SSA, CCA, and untreated SSA with or without inoculation of bacteria. [file 13068_2016_480_MOESM4_ESM.pdf]
