# Supplementary material for: Enhanced electrical power generation using flame-oxidized stainless steel anode in microbial fuel cells and the anodic community structure
Source: Biotechnol Biofuels. 2016 Mar 12;9:62. doi: 10.1186/s13068-016-0480-7 (PMC4788886; doi:10.1186/s13068-016-0480-7)
Supplement: Supplementary file 3 — 10.1186/s13068-016-0480-7 Time courses of electricity generation in MFCs equipped with FO-SSA or CCA. [file 13068_2016_480_MOESM3_ESM.pdf]

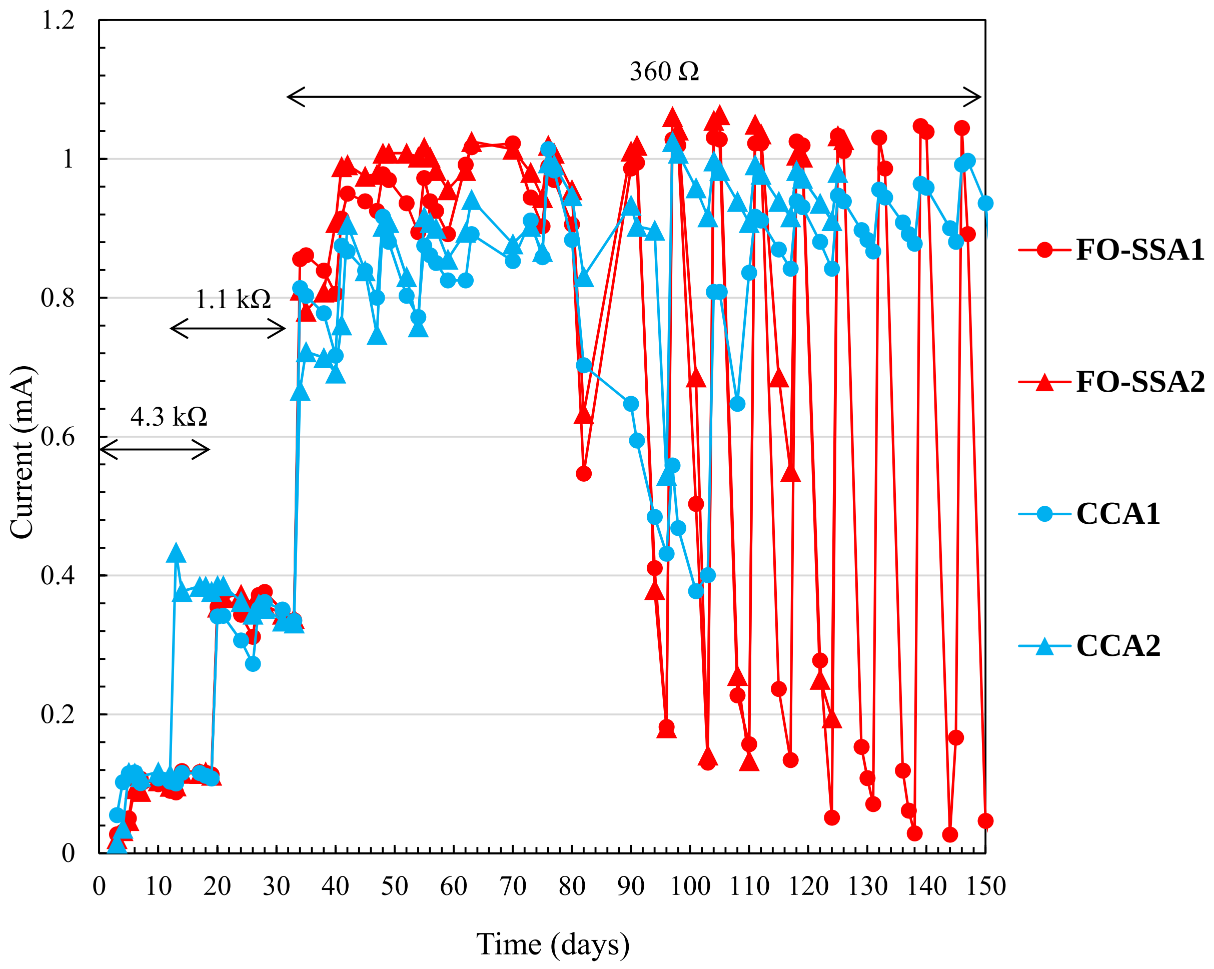

Figure S3. Time courses of electricity generation in MFCs equipped with FO-SSA or CCA. The MFCs were operated at 25˚C in a fed-batch mode at a medium-exchange interval of 7 days using the peptone medium. The MFCs were connected to a 4.3 kΩ external resistor, and the resistance value was decreased stepwise to 1.1 kΩ and 360 Ω during operation.
