# Supplementary material for: Enhanced electrical power generation using flame-oxidized stainless steel anode in microbial fuel cells and the anodic community structure
Source: Biotechnol Biofuels. 2016 Mar 12;9:62. doi: 10.1186/s13068-016-0480-7 (PMC4788886; doi:10.1186/s13068-016-0480-7)
Supplement: Supplementary file 5 — 10.1186/s13068-016-0480-7 Number of reads after chimera check and alpha diversity analysis of anodic communities in MFCs. [file 13068_2016_480_MOESM5_ESM.pdf]

Table S1. Number of reads after chimera check and alpha diversity analysis of anodic communities in MFCs

Sample	No. of reads	No. of OTUs	Chao1 richness	ACE	Shannon's diversity index	Good's coverage
FO-SSA5	363,447	2,458	3,360	3,515	5.759	0.9977
FO-SSA6	435,412	2,871	3,670	3,920	5.384	0.9978
CCA3	705,165	3,554	4,414	4,678	5.009	0.9985
SSA3	533,460	2,437	3,180	3,416	4.760	0.9984
FO-SSA7-o.c	849,173	3,034	3,258	3,470	5.307	0.9993
AS	1,441,604	11,148	11,158	11,246	9.033	0.9998
