# Supplementary material for: Enhanced electrical power generation using flame-oxidized stainless steel anode in microbial fuel cells and the anodic community structure
Source: Biotechnol Biofuels. 2016 Mar 12;9:62. doi: 10.1186/s13068-016-0480-7 (PMC4788886; doi:10.1186/s13068-016-0480-7)
Supplement: Supplementary file 6 — 10.1186/s13068-016-0480-7 Rarefaction curves (A) and PCo plot (B) showing the relationship among the bacterial communities of the anodic biofilms and AS inoculated into the MFCs. [file 13068_2016_480_MOESM6_ESM.pdf]

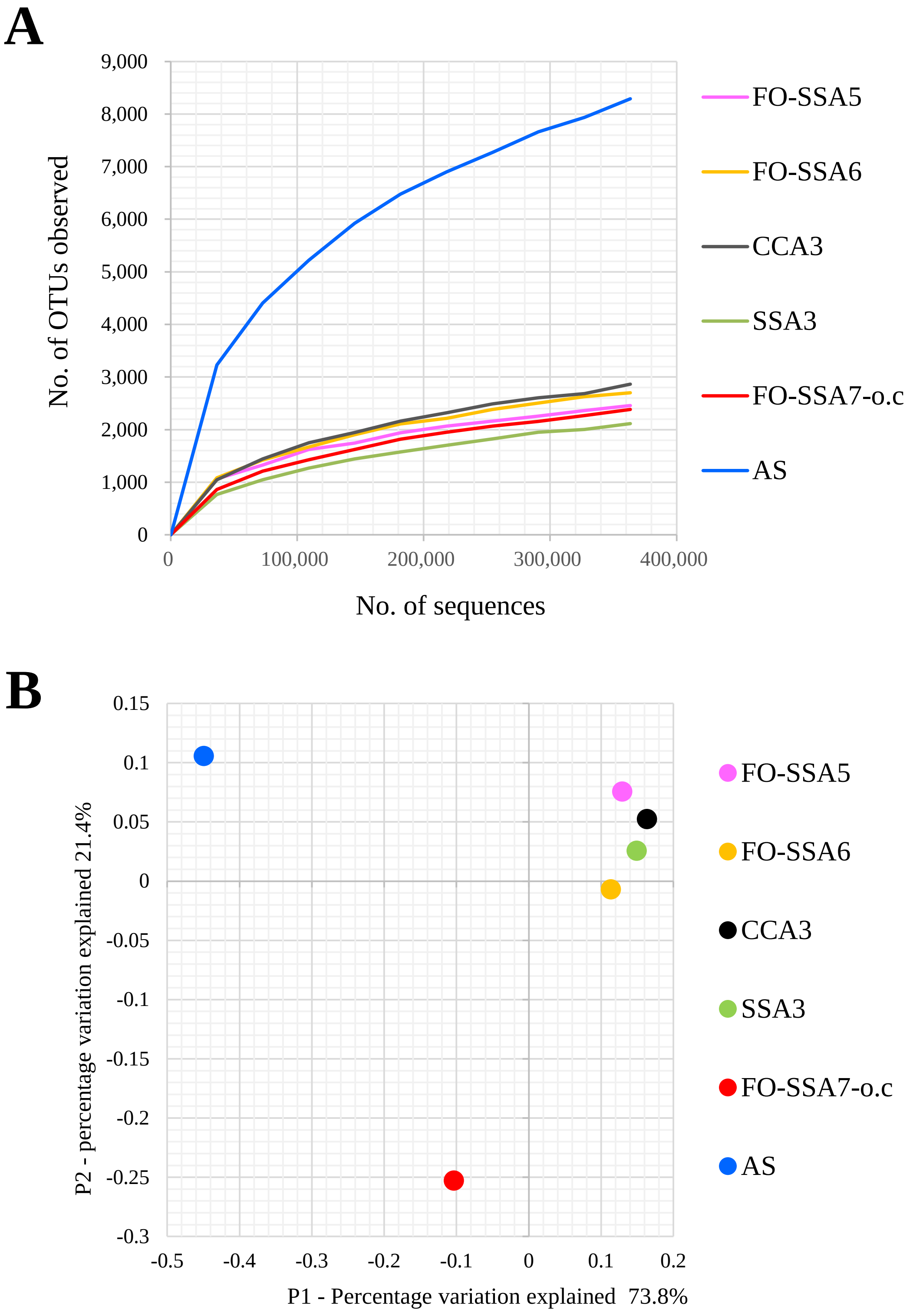

Figure S5. Rarefaction curves (A) and PCo plot (B) showing the relationship among the bacterial communities of the anodic biofilms and AS inoculated into the MFCs.
