# Supplementary material for: Enhanced electrical power generation using flame-oxidized stainless steel anode in microbial fuel cells and the anodic community structure
Source: Biotechnol Biofuels. 2016 Mar 12;9:62. doi: 10.1186/s13068-016-0480-7 (PMC4788886; doi:10.1186/s13068-016-0480-7)
Supplement: Supplementary file 7 — 10.1186/s13068-016-0480-7 Genera detected in the biofilms of FO-SSA5 and FO-SSA6 with higher abundance than those in FO-SS7-o.c. [file 13068_2016_480_MOESM7_ESM.pdf]

Table S2. Genera detected in the biofilms of FO-SSA5 and FO-SSA6 with higher abundance than those in FO-SS7-o.c

Phylum	Genus	FO-SSA7-o.c	FO-SSA5	FO-SSA6	CCA3	SSA3	AS
Synergistetes	*vadinCA02*	1.49% (1.0)	37.9% (25.4)	32.5% (21.8)	46.1% (30.9)	40.1% (26.9)	0.05% (<0.1)
Proteobacteria	*Geobacter*	1.43% (1.0)	8.85% (6.2)	9.16% (6.4)	1.40% (1.0)	0.65% (0.5)	0.01% (<0.1)
Bacteroidetes	Blvii28	0.00% (1.0)	4.08% (>100)	3.20% (>100)	1.04% (> 100)	3.22% (> 100)	0.01% (2.5)
Synergistetes	PD-UASB-13	0.00% (1.0)	1.87% (>100)	0.69% (>100)	1.68% (>100)	1.72% (>100)	0.00% (0.3)
Armatimonadetes	RB046	0.00% (1.0)	0.51% (77.9)	1.47% (>100)	0.21% (31.9)	0.17% (26.0)	0.00% (0.5)
Chlorobi	OPB56	1.0 (0.02%)	0.57% (37.3)	1.09% (70.4)	0.75% (48.8)	1.7% (>100)	0.01% (0.8)
Firmicutes	*Syntrophomonas*	0.01% (1.0)	1.03% (98.7)	0.54% (51.3)	0.17% (16.5)	0.18% (17.4)	0.00% (0.1)
Spirochaetes	*Treponema*	0.03% (1.0)	0.61% (21.0)	0.54% (18.5)	0.51% (17.5)	0.48% (16.5)	0.02% (0.6)
Firmicutes	*Clostridium*	0.14% (1.0)	0.59% (4.1)	0.54% (3.6)	0.47% (3.3)	0.38% (2.6)	0.12% (0.9)

The detection frequency of genera in the biofilms binding to FO-SSA, CCA, SSA, and FO-SSA with an open circuit are expressed as percentage. For comparison, the frequency in AS, inoculated into the MFCs, is also shown. The values in parenthesis indicate the multiple numbers of the frequencies to FO-SSA with an open circuit.
