# Supplementary material for: Enhanced electrical power generation using flame-oxidized stainless steel anode in microbial fuel cells and the anodic community structure
Source: Biotechnol Biofuels. 2016 Mar 12;9:62. doi: 10.1186/s13068-016-0480-7 (PMC4788886; doi:10.1186/s13068-016-0480-7)
Supplement: Supplementary file 8 — 10.1186/s13068-016-0480-7 Atomic composition of SS used in this study. [file 13068_2016_480_MOESM8_ESM.pdf]

Table S3. Atomic composition of SS used in this study

	Si	P	S	Cr	Mn	Fe	Ni	Cu
SS (SUS304) mesh	0.38	0.03	0.05	17.98	1.72	69.27	10.21	0.37

Chemical composition, with the exception of the oxygen atom, was analyzed by XRF. The values indicate weight% of atoms
